# Exercise in CKM syndrome progression: a stage-specific approach to cardiovascular, metabolic, and renal health

**DOI:** 10.1186/s12933-025-03029-4

**Published:** 2025-12-20

**Authors:** Zacharias Papadakis

**Affiliations:** Department of Health Sciences and Clinical Practice, College of Health Professions and Medical Sciences, Barry University, 11300 NE 2nd Ave, Miami Shores, FL 33161 USA

**Keywords:** Moderate-intensity continuous exercise, High-intensity interval exercise, Resistance training, Sex differences in exercise adaptation

## Abstract

Cardiovascular-Kidney-Metabolic (CKM) syndrome progresses through distinct stages, from early metabolic risk factors to advanced cardiovascular disease and kidney dysfunction. Across these stages, exercise remains a central yet underutilized intervention, offering physiological adaptations that address metabolic dysregulation, vascular dysfunction, and inflammation. This review evaluates the stage-specific effects of moderate-intensity continuous exercise (MICE), high-intensity interval exercise (HIIE), and resistance training (RT) on CKM syndrome. In Stage 0, exercise augments insulin sensitivity, endothelial function, and mitochondrial biogenesis, preserving optimal health in individuals without metabolic risk factors. Stage 1, marked by excess adiposity, sees structured exercise regimens effectively reducing visceral fat, improving lipid profiles, and enhancing glucose regulation. During Stage 2, encompassing metabolic risk factors and early chronic kidney disease (CKD), aerobic and resistance exercise improve endothelial responsiveness, glycemic control, and renal outcomes. In Stage 3, subclinical cardiovascular disease, targeted exercise interventions strengthen vascular integrity, boost cardiac efficiency, and enhance metabolic resilience. Finally, Stage 4 entails clinical CVD, where exercise-based rehabilitation programs (e.g., moderate-intensity continuous training [MICT], high-intensity interval training [HIIT]) raise functional capacity, improve quality of life, and support favorable prognosis. Sex differences in exercise adaptations underscore the importance of individualized prescriptions. Emphasizing a multidisciplinary strategy that integrates lifestyle modifications and clinical measures can mitigate CKM-associated morbidity and mortality. Future research should investigate long-term exercise adherence, sex-specific responses, and the role of digital health tools to optimize CKM management and patient outcomes.

## Key points

This manuscript explains how Cardiovascular-Kidney-Metabolic (CKM) syndrome progresses from having no risk factors (Stage 0) to serious heart disease and kidney problems (Stage 4). Each stage benefits from different exercise approaches, like moderate-intensity continuous workouts, high-intensity interval sessions, and resistance training, to reduce inflammation, boost blood flow, and improve metabolism.

Because men and women often respond differently to exercise, and because factors like obesity or kidney disease can change how effective workouts are, personalized exercise plans are important. A “one-size-fits-all” approach may miss the unique needs of each patient.

Long-term success requires better ways to keep people active and engaged, including digital tools and coaching. By focusing on individual needs and regularly adjusting exercise routines, healthcare teams can help reduce the overall impact of CKM syndrome.

## Introduction

Cardiovascular-Kidney-Metabolic (CKM) syndrome is a multisystem disorder characterized by the interrelated pathophysiology of cardiovascular disease (CVD), chronic kidney disease (CKD), and metabolic dysfunction (e.g., obesity, insulin resistance, dyslipidemia). These conditions interact bidirectionally, exacerbating disease burden and increasing morbidity and mortality [1, 2]. As CKM syndrome progresses from Stage 0 (absence of risk factors) to Stage 4 (clinical CVD with or without kidney failure), inflammation, oxidative stress, endothelial dysfunction, and metabolic dysregulation accelerate disease onset and severity [2–6].

Despite pharmacological advancements, structured exercise programs remain underutilized in CKM management, despite their proven benefits. Exercise enhances mitochondrial function, endothelial health, and insulin sensitivity while reducing visceral adiposity, systemic inflammation, and metabolic risk factors, establishing its role as a cornerstone of CKM care [6–15]. Evidence suggests that exercise efficacy varies across CKM stages, requiring a tailored, stage-specific approach that accounts for disease progression, comorbidities, and physiological responses [1, 2, 16–23]. However, efficacy is fundamentally limited by long-term patient adherence, which remains a core barrier to mitigating CKM progression in clinical practice [24, 25].

This review expands on previous work, which examined lifestyle medicine interventions, including physical activity (PA) and exercise (PE) in CKM syndrome [26]. Here, we focus on stage-specific exercise interventions, evaluating the effects of moderate-intensity continuous exercise/training (MICE/MICT), high-intensity interval exercise/training (HIIE/HIIT), and resistance exercise/training (RE/RT) on CKM progression. Given the heterogeneity of exercise responses, modulated by sex, adiposity, and disease state, this review highlights the necessity of personalized exercise prescriptions to optimize CKM outcomes [17, 27–39].

By synthesizing findings from randomized controlled trials (RCTs) and meta-analyses, this narrative review bridges research and clinical practice, advocating for a multimodal, individualized approach to CKM exercise interventions. Future directions should prioritize strategies to improve long-term adherence, sex-specific adaptations, and the integration of digital health technologies to enhance patient engagement and clinical implementation, an endeavor that requires reversing the trend of defunding precision lifestyle medicine to overcome the CKM crisis [8, 33, 35, 36, 39–45]. Emerging wearable-sensor methods to quantify psychological readiness [46] and replicate robust experimental designs may accelerate that effort [47].

## Stage 0: no CKM health risk factors

Stage 0 encompasses individuals without metabolic risk factors, CKD, or subclinical/clinical CVD [1]. In this population, regular physical activity and structured exercise play a critical role in maintaining physiological homeostasis, promoting long-term health, and reducing the risk of chronic disease [7, 40, 48, 49].

Current PA guidelines recommend 150–300 min of moderate-intensity aerobic activity or 75–150 min of vigorous-intensity activity per week, complemented by regular muscle-strengthening exercises for optimal health outcomes [8, 40]. Additionally, engaging in at least 60 min of moderate-to-vigorous activity daily supports weight regulation and cardiometabolic health [50, 51]. Individual responses to exercise vary, influenced by the interplay between energy expenditure and intake, reinforcing the need for personalized activity plans [8, 40].

Regular exercise enhances cardiorespiratory fitness by improving maximal oxygen uptake (VO₂max) and oxygen utilization efficiency and promotes tighter autonomic regulation even under disrupted sleep [19, 52]. Taken together, these adaptations extend beyond metabolism to vascular and inflammatory pathways, improving insulin sensitivity, facilitating glucose uptake in skeletal muscle, and modulates lipid metabolism, lowering triglyceride levels while increasing high-density lipoprotein (HDL) cholesterol [3, 4, 16, 48, 53]. Beyond metabolic benefits, exercise supports vascular endothelial function, optimizing vasodilation and blood flow regulation [48, 54]. Additionally, it reduces chronic low-grade inflammation, a key contributor to CKD and CVD progression [48, 55]. At the cellular level, exercise stimulates mitochondrial biogenesis, improving ATP production and metabolic efficiency [48, 56]. Both aerobic and resistance exercise enhance muscle strength, endurance, and overall functional capacity. Sustained endurance training further boosts VO₂max, a primary predictor of longevity and cardiovascular health [28, 57–61], with comparable network-level adaptations observed in performance settings [62].

These physiological adaptations highlight the preventive and performance-enhancing role of exercise in individuals without CKM risk factors. By fostering long-term resilience and metabolic efficiency, regular physical activity remains an essential component of overall health maintenance (Table 1).

**Table 1:** Stage-specific goals and primary effects of exercise in CKM syndrome (qualitative summary; see Table 2 for typical magnitudes and minimum effective durations)

CKM stage	Primary clinical goals	Lead training modalities	Principal exercise-induced effects (qualitative, exemplar evidence)
Stage 1—Excess/dysfunctional adiposity & early metabolic risk.	Prioritize adverse fat depots (visceral, hepatic), improve insulin sensitivity, glycemic control, and early vascular health.	MICT (150–300 min/wk), HIIT (interval formats), RT 2–3 d/wk; combined training when feasible.	↓ hepatic fat with both HIIT and MICT (8, 40, 50, 51, 63, 64); modest weight/fat loss with exercise alone vs. energy restriction/AOMs (40, 65); early improvement in vascular indices possible (umbrella/meta-analytic signals) (57, 58, 66, 67).
Stage 2—Dysglycemia/T2D and emerging vascular dysfunction.	Tighten glycemic control; enhance endothelial function; reduce arterial stiffness; raise CRF.	MICT, HIIT, RT; combined training for glycemia + vascular targets.	↑ flow-mediated dilation in T2D with training(67); ↑ endothelial function and ↓ arterial stiffness in older adults (66); stage framing aligned to AHA CKM pathophysiology (1, 2).
Stage 3—Subclinical CVD.	Improve CRF, autonomic/vascular function; consolidate risk-factor control; prepare for secondary prevention pathways.	Interval-based aerobic + MICT; add RT; consider combined training.	↑ exercise capacity post-MI with interval formats (68–70); RT complements aerobic training in CVD (71).
Stage 4—Clinical CVD (e.g., CAD, HF, PAD).	Secondary prevention; symptom relief; functional capacity; event risk reduction in supervised programs.	Cardiac rehab framework: MICT or interval-aerobic + RT; PAD: supervised/home-based walking to near-max claudication.	Combined aerobic + RT is advantageous in CR programs (position/consensus)(71); HFpEF benefits from supervised training (72); PAD: supervised treadmill and home-based walking improve 6MWD and treadmill performance over 12–24 wk (73, 74).

Abbreviations: CKM, cardiovascular-kidney-metabolic; MICT, moderate-intensity continuous training; HIIT, high-intensity interval training; RT, resistance training; CRF, cardiorespiratory fitness; CR, cardiac rehabilitation; PAD, peripheral artery disease; AOMs, anti-obesity medications; 6MWD, 6 min walk distance; CVD, cardiovascular disease; CAD, coronary artery disease; HF, heart failure; HFpEF, heart failure with preserved ejection fraction

## Stage 1: Excess and/or dysfunctional adiposity

Stage 1 of CKM syndrome includes individuals with overweight or obesity or those with dysfunctional adipose tissue but without metabolic risk factors or CKD [1]. This classification is based on a body mass index (BMI) ≥ 25 kg/m² (≥ 23 kg/m² for Asian ancestry), a waist circumference ≥ 88 cm in women or ≥ 102 cm in men (≥ 80 cm and ≥ 90 cm, respectively, for Asian ancestry), or fasting blood glucose levels of 100–124 mg/dL or hemoglobin A1c (HbA1c) between 5.7% and 6.4% [1]. A consolidated, system-level synopsis of carbohydrate/lipid metabolism and body-composition effects appears in Sect. 7.

Exercise interventions, including both aerobic and resistance training, play a critical role in managing excess adiposity and insulin resistance. Aerobic exercise improves insulin sensitivity by enhancing glucose uptake and utilization in skeletal muscle, offering a protective mechanism against insulin resistance [5, 63]. In addition, reductions in visceral fat, a key determinant of cardiometabolic risk, are a principal benefit of aerobic training [64]. A study of young women in NCAA-compliant preseason programs further document favorable shifts in whole-body composition [65].

Exercise further modulates lipid metabolism by lowering triglyceride levels, an effect influenced by exercise intensity and excess post-exercise oxygen consumption, while raising HDL cholesterol and improving blood pressure, thereby mitigating inflammation and insulin resistance [64, 66]. Nutritional strategies, such as probiotic supplementation, may complement structured exercise regimens by enhancing cardiorespiratory endurance and facilitating visceral fat reduction [5, 64, 67–73].

A longitudinal study of 406 adults (aged 35–70 years) evaluated the impact of resistance training, aerobic exercise, and combined modalities on CVD risk factors over one year. Participants who engaged in supervised, one-hour sessions three times per week achieved greater improvements in composite CVD risk scores, underscoring exercise’s role in metabolic health [56]. Similarly, combined (i.e., defined as when distinct resistance and endurance training are concurrently performed in the same exercise session) [74] and hybrid training models (i.e., defined as a multicomponent exercise during which the cardiovascular and musculoskeletal systems are engaged simultaneously at various intensities throughout a single session) [75, 76] demonstrated cardiometabolic benefits, with sex differences influencing outcomes [8].

Exercise intensity significantly affects physiological adaptations. A 12-week study reported that high-intensity training produced the most pronounced gains in aerobic capacity, body mass reduction, and fitness metrics, while varying intensity levels promoted enhanced muscle strength via improved neural recruitment and motor unit activation [27]. Additionally, aerobic training significantly improved vascular function by increasing flow-mediated dilation (FMD) in children and adolescents and reducing pulse wave velocity (PWV) and intima-media thickness (IMT) across age groups. In contrast, resistance training had a more moderate effect on arterial stiffness [77]. Consistent with these modality and intensity responses, a meta-analysis of RCTs investigating exercise effects in overweight and obese individuals with type 2 diabetes, hypertension, dyslipidemia, metabolic syndrome, and non-alcoholic fatty liver disease (NAFLD)/non-alcoholic steatohepatitis (NASH) revealed notable decreases in insulin resistance, as measured by improved homeostatic model assessment of insulin resistance (HOMA-IR) [78]. Beyond visceral adiposity, ectopic fat depots, particularly intrahepatic triglyceride (hepatic steatosis), are pathophysiologically distinct and clinically salient in CKM/MetS. Furthermore, both HIIE and MICE are effective in reducing ectopic fat stores, such as hepatic fat, a key driver of metabolic dysfunction in Stage 1, an outcome that is often achieved without mandated dietary change [2, 79]. Both HIIE and MICE also influenced appetite regulation, with significant reductions in ghrelin levels observed in obese men, suggesting potential benefits for appetite suppression and weight management [80].

Adipokines such as leptin and adiponectin also play key roles in metabolic dysfunction. The “leptin paradox” indicates leptin resistance as a primary driver of arterial stiffness, whereas the “adiponectin paradox” points to impaired clearance, rather than increased production, as a factor in adverse metabolic outcomes [81]. Collectively, these findings underscore the complex pathophysiology of obesity-related cardiovascular dysfunction and highlight the importance of individualized exercise prescriptions for Stage 1 CKM syndrome.

A well-established intervention to improve cardiometabolic health in overweight and obese populations is MICE. Typically performed 2–4 days per week at 60–75% of maximum heart rate (HRmax) or 50–70% of heart rate reserve (HRR), MICE has demonstrated improvements in endothelial function and reductions in metabolic risk factors. For instance, a study of middle-aged adults (BMI >24.9 kg/m²) who engaged in three 60-minute treadmill sessions weekly at 50–75% HRmax (~ 500 kcal per session) showed significant decreases in LDL and total cholesterol levels, along with beneficial changes in body fat percentage (BF%) and HDL cholesterol [82].

Enhancements in endothelial function following MICE are linked to increased nitric oxide (NO) bioavailability, facilitating vasodilation and optimal blood flow regulation. Comparative evidence indicates that MICE may offer longer-lasting FMD improvements than HIIE, emphasizing its potential for sustained vascular benefits [77, 83].

Moderate-intensity continuous exercise can reduce subcutaneous adiposity and improve glucose metabolism; however, weight loss attributable to exercise alone is typically modest (~ 0.5–3.0 kg) and smaller than losses achieved with energy-restricted diets and contemporary anti-obesity pharmacotherapy [40, 84], making it a viable option for individuals who may find high-intensity protocols challenging [25, 40, 85, 86]. However, while short-term aerobic training alleviates arterial stiffness, these benefits may diminish in older adults without ongoing participation [87]. These vascular and metabolic gains are transient; when training stops, improvements in arterial stiffness, FMD, and insulin sensitivity wane, underscoring the need for continued, structured exercise rather than short blocks of training [87, 88].

Both MICE and HIIE yield positive cardiometabolic outcomes, although HIIE often leads to quicker gains in VO₂max and body composition, whereas MICE provides steadier benefits over extended periods [88, 89]. Consequently, individualized exercise prescriptions remain essential, taking into account each participant’s fitness level, adherence capacity, and specific health objectives.

High-intensity interval training is a time-efficient and highly effective method for enhancing cardiovascular and metabolic health in overweight and obese populations. Characterized by brief, high-intensity bouts (85–95% HRmax) interspersed with lower-intensity recovery, HIIT engages both aerobic and anaerobic energy systems. Studies show that HIIT can improve vascular function by increasing FMD by 3.9% and arterial diameter by 4.8% compared to MICT [90–92]. These vascular adaptations are attributed to greater shear stress, elevated NO bioavailability, and endothelium-dependent vasodilation [77].

High-intensity interval training’s metabolic benefits include higher rates of visceral fat reduction and increased catecholamine secretion, supporting waist circumference reduction and fat oxidation [93]. Systematic reviews also reveal that HIIT confers superior improvements in VO₂max, fasting blood glucose, and total cholesterol compared to MICT, underlining its value in cardiometabolic health [88].

While HIIT’s shorter duration may boost adherence for some, its intensity can pose challenges for individuals with lower fitness levels or musculoskeletal concerns [91]. Despite substantial gains in peak oxygen uptake (VO₂peak) and mitochondrial function, HIIT does not outperform MICE in reducing overall BF% among people with excess weight [94].

These findings suggest that HIIT offers a promising option for achieving rapid cardiovascular and metabolic gains, although exercise programs should be tailored to accommodate individual tolerance, fitness status, adherence capacity, and complex psychosocial profiles to ensure sustainability [95, 96, 97]. examine the long-term impact of HIIT on diverse populations, particularly regarding variations in individual responses and potential risks associated with high-intensity protocols.

Research into muscle-kidney crosstalk, mediated by exercise-induced cytokines (“exerkines”) and muscle-secreted growth factors, elucidates resistance training’s broader benefits, suggesting therapeutic avenues for CKM syndrome management via targeted exercise for muscle/vascular health [16, 30, 98, 99]. Resistance training is a powerful modality for improving body composition, muscle strength, and metabolic health, largely through increases in lean mass that boost basal metabolic rate and insulin sensitivity. In one study featuring thrice-weekly, eight-week RT sessions (six exercises, 3 sets of 12 reps at 50–75% 1-RM), participants significantly lowered body fat percentage, LDL, and total cholesterol while increasing HDL cholesterol [82]. Both low- and high-volume RT protocols have similarly reduced total and abdominal fat, glucose levels, and C-reactive protein (CRP), underscoring RT’s role in mitigating metabolic inflammation [100]. However, RT alone may be less effective than aerobic exercise in lowering overall BMI and fat mass, highlighting the importance of blended training strategies [89]. It also supports vascular function, though its effects on endothelial health are less established than those of aerobic modalities. Some studies link RT to improvements in FMD, PWV, and IMT, but findings remain inconclusive due to methodological variations [77, 91].

Multimodal exercise routines, combining resistance and aerobic training, confer the most extensive metabolic benefits. For example, RT paired with moderate-intensity aerobic exercise (40–80 min, 5 times per week) has been shown to enhance FMD, PWV, IMT, LDL-C, fasting insulin, and HOMA-IR, while reducing triglycerides [89]. These findings underscore the need to incorporate RT into structured exercise programs, particularly for those seeking to bolster metabolic resilience and maintain muscle mass during weight loss.

Sex differences significantly influence exercise adaptations, affecting fat distribution, hormonal regulation, and cardiometabolic risk. For example, total-body composition explains addition cardiorespiratory fitness across age and sex, supporting tailored cut-points [101]. These variations underscore the importance of sex-specific exercise prescriptions for effective CKM management. Moderate-intensity continuous exercise tends to enhance insulin sensitivity and reduce visceral adiposity more effectively in women, whereas men experience greater central adiposity reductions from high-intensity exercise [27, 64, 102]. Due to their predominance of subcutaneous fat storage, women generally benefit more from MICE-induced increases in fat oxidation [99], while men’s higher visceral fat storage corresponds to better HIIT responses [27, 64, 102]. Resistance training benefits both sexes, but men typically exhibit larger hypertrophy gains due to testosterone-driven anabolic mechanisms [103].

Moreover, women often display superior endurance adaptations and flexibility improvements, whereas men achieve more pronounced gains in muscle strength and power [104, 105]. Premenopausal women’s higher subcutaneous fat stores can confer cardioprotective advantages, while men’s greater visceral fat accumulation elevates cardiometabolic risk [106, 107]. Recognizing these differences is essential for customizing exercise recommendations to optimize physiological outcomes and ensure sustained adherence in both sexes [54, 64, 108].

## Stage 2: Metabolic risk factors and CKD

Stage 2 of CKM syndrome includes individuals with metabolic risk factors such as hypertriglyceridemia, hypertension, metabolic syndrome (MetS), type 2 diabetes mellitus (T2DM), or CKD [1]. Regular physical activity and structured exercise are vital for managing cardiovascular and renal risks, offering both primary and secondary protection against CKD progression [1, 109, 110]. Nevertheless, exercise remains underutilized in CKM populations [111]. For a consolidated summary of glycemic, vascular, and renal outcomes across modalities, see Sect. 7.

Multiple exercise modalities, including aerobic training, RT, and HIIE, influence glucose regulation, with effects contingent on intensity, duration, and timing [112]. In T2DM management, aerobic exercise has demonstrated notable reductions in systemic inflammation (CRP, IL-6, resistin, TNF-α), coupled with improvements in glycemic control and fat mass [102]. Supervised aerobic exercise appears particularly effective at lowering systemic inflammation, though changes in adiponectin remain inconsistent [113].

Improved kidney blood flow and reduced inflammation underlie the renal-protective benefits of exercise, potentially slowing CKD progression [55]. Acute aerobic sessions in moderate CKD transiently lower serum creatinine and raise eGFR without compromising filtration [18]. A parallel study showed the same bout favorably resets heart-rate variability in non-dialysis CKD [19]. Moreover, exercise mitigates muscle atrophy, reduces albuminuria, and does not exacerbate proteinuria in CKD patients [14, 111]. Across sexes, regular exercise positively impacts blood pressure, lipid profiles, and overall cardiometabolic health [5].

Exercise interventions help lower systolic and diastolic blood pressure, increase muscle mass and endurance, enhance aerobic capacity, and improve physical function [35, 110]. Notably, hypertensive individuals may exhibit heightened inflammatory responses to acute exercise relative to normotensive peers [114]. A meta-analysis of 84 RCTs (5,065 participants) confirmed that exercise lowers systolic and diastolic blood pressure irrespective of modality [115]. In women with polycystic ovary syndrome (PCOS), MICT and HIIE both enhance cardiorespiratory fitness (CRF) and metabolic parameters, although HIIE may yield superior insulin sensitivity and hormonal outcomes [116].

A comparison of HIIE and MICE in mid-stage CKD reported improved endothelial responsiveness and reduced oxidative stress markers (e.g., asymmetric dimethylarginine [ADMA]) for both, with MICE uniquely elevating antioxidant levels such as paraoxonase-1 (PON1) and total antioxidant capacity (TAC) [117]. Furthermore, acute aerobic exercise in moderate CKD lowers serum creatinine (sCr) and increases eGFR, implying enhanced renal function [18].

Research contrasting HIIE and RT has revealed distinct post-exercise metabolic responses, including differences in glucose and cortisol levels [118]. At low-to-moderate loads, RT effectively reduces fasting blood glucose, supporting MetS management [31, 89, 109]. Exercise also augments endothelial function, NO bioavailability, and overall cardiometabolic profiles, reinforcing its importance for managing hypertension and anticipating its future development, which is critical for renal health [119, 120].

For individuals with Stage 2 CKM syndrome, exercise remains central to mitigating metabolic and cardiovascular risks, particularly when paired with dietary interventions like the DASH diet [29]. However, tailoring exercise prescriptions to population-specific lipid profiles and other individualized factors remains critical [16].

Moderate-intensity continuous exercise is widely advocated for individuals with CKD, T2DM, and other cardiometabolic conditions. Current guidelines recommend a minimum of 150 min of moderate-intensity aerobic activity weekly to bolster cardiovascular, metabolic, and renal health [111]. A step goal of 7,000–10,000 steps per day is generally advised, although ~ 5,000 daily steps may be more feasible for CKD patients [111]. It enhances body composition, lowers blood pressure, and increases insulin sensitivity, making it integral to metabolic disease management [109]. In T2DM, moderate-intensity aerobic training (AT) reduces body weight, visceral fat, and blood pressure while improving glucose uptake and glycemic control [109]. Combining RT with MICE further preserves lean mass during weight loss and augments metabolic adaptations [109].

By diminishing vasoconstrictors such as endothelin-1 and angiotensin II, MICE reduces arterial stiffness, improves endothelial function, and optimizes blood flow regulation [119]. These vascular benefits lower PWV and augmentation index (AIx), mitigating hypertension and cardiovascular strain, particularly among CKD patients [119].

When performed 3–7 times per week at light-to-moderate intensity (60–85% HRR or VO₂peak) for 30–60 min per session, MICE significantly boosts lipid profiles, BMI, and overall metabolic health [31]. For hypertension management, pairing moderate-intensity aerobic exercise with low-to-moderate-load RT yields maximal effects on blood pressure, LDL reduction, and BMI regulation [31].

While short-term MICE alleviates arterial stiffness, these gains may wane without sustained participation, particularly among older adults [87]. Nonetheless, incorporating aerobic exercise into CKM care plans provides valuable cardiovascular, metabolic, and renal advantages, underscoring the importance of individualized exercise prescriptions tailored to each patient’s health status and functional capacity [111].

High-intensity interval training is an efficient and effective strategy for enhancing cardiometabolic health, particularly in individuals with CKD, T2DM, and MetS. Requiring as little as 75 min of exercise weekly, compared to 150 min for MICE, HIIT can deliver comparable or superior benefits [109]. Characterized by short intervals of high-intensity effort (85–95% HRmax) followed by lower-intensity recovery, HIIT engages both aerobic and anaerobic pathways.

In a 16-week supervised program, HIIT significantly improved MetS markers, including VO₂max, lipid profiles, and insulin sensitivity, with participants completing three weekly 43-minute sessions [29]. A systematic review of 23 RCTs also reported that low-volume HIIT yields benefits similar to MICE but confers greater improvements in total cholesterol, HDL-C, LDL-C, and triglycerides [121]. For individuals with T2DM, HIIT has demonstrated positive effects on flow-mediated dilation (FMD), endothelial function, and NO bioavailability [122]. Likewise, a 12-week concurrent HIIT and resistance training study saw improvements in VO₂ peak, O₂ pulse, and ventilatory threshold (VT1) across all groups, with T2DM participants showing marked reductions in cardiovascular risk and metabolic dysfunction [63].

Moreover, HIIT also significantly improves glycemic markers, including HbA1c, fasting blood glucose, fasting blood insulin, and HOMA-IR [52], and waist circumference [123]. However, it does not substantially affect fat-free mass, and glycemic outcomes can vary by protocol design and individual metabolic responses [113]. Moreover, the cardiometabolic benefits of HIIT can be modulated by lifestyle factors, as acute partial sleep deprivation has been shown to blunt post-exercise improvements in endothelial function and alter cardiac autonomic modulation following a HIIT session [52, 124].

In women with PCOS, HIIT enhances insulin sensitivity and elevates sex hormone-binding globulin (SHBG) levels, offering potential hormonal regulation benefits [116]. Nevertheless, for some PCOS patients, MICE may provide similar or even superior gains in cardiorespiratory fitness, underscoring the need for tailored interventions [125].

While HIIT is associated with improved cardiometabolic outcomes, its intensity may impede adherence among sedentary individuals or those with preexisting cardiovascular risk [91]. An umbrella review indicates that HIIT can bolster participant enjoyment and motivation, which may foster long-term adherence [121, 126].

Medical clearance is advisable before initiating HIIT in those with metabolic diseases, especially when cardiovascular risk is high [24]. Until more robust data emerge on its sustained efficacy for diabetes management, moderate aerobic exercise and post-meal resistance training remain favored for glycemic control [113]. Overall, HIIT is a potent option for improving cardiovascular, metabolic, and hormonal health, although it should be customized to individual fitness levels, tolerances, and long-term adherence potential.

Resistance training is pivotal for improving muscle strength, body composition, metabolic health, and cardiovascular function in individuals with CKD, T2DM, and MetS. Although RT alone may not result in substantial weight loss without caloric restriction, it effectively lowers fat mass (including abdominal fat) and increases lean mass, boosting basal metabolic rate and insulin sensitivity [109]. By improving glycemic control and reducing fasting glucose, RT serves as a potent strategy for T2DM management and prevention [109].

Consistent RT correlates with lower HbA1c, enhanced glucose tolerance, and reduced total cholesterol and triglyceride levels, especially among older adults and those with prediabetes or T2DM [35, 109, 127]. Beyond metabolic benefits, RT positively impacts lipid profiles and decreases hypertension risk in CKM populations [109]. For example, RT at low-to-moderate intensities significantly improved fasting glucose and lipid metabolism in MetS [31].

Resistance training drives muscle hypertrophy, strength, and endurance, crucial for averting sarcopenia and preserving functional capacity in CKD patients [127]. It can also bolster oxidative enzyme activity and shift muscle fiber composition, supporting cardiovascular health over the long term [127].

While aerobic training more effectively reduces BMI and overall fat mass, RT contributes markedly to endothelial function, NO bioavailability, and arterial compliance, thus lowering blood pressure. However, current data on RT’s direct endothelial effects remain inconclusive due to study heterogeneity [91, 119].

It seems that RT is generally most beneficial when performed 2–3 times weekly, targeting major muscle groups with progressive overload. High-intensity RT (≥ 70% 1-RM) is especially advantageous for glucose regulation, whereas low-to-moderate intensity (50–70% 1-RM) is advised for those with heightened cardiovascular risk [112]. Combining RT with aerobic exercise (MICE or HIIT) leads to superior gains in glycemic control, lipid metabolism, and overall cardiometabolic health compared to either approach alone [112].

Studies show that integrating resistance and aerobic exercise lowers HbA1c, boosts insulin sensitivity, and enhances weight loss outcomes [35]. Thus, RT stands as a key intervention for cardiometabolic disease management, yielding distinct benefits in strength, metabolic function, and lipid regulation. While aerobic exercise remains optimal for broad fat reduction, RT is crucial for maintaining lean mass, mitigating sarcopenia, and promoting favorable long-term outcomes in CKD and T2DM. Overall, a blend of aerobic and resistance modalities maximizes metabolic and cardiovascular adaptations. Gains in glycemic control, blood pressure, and renal vascular function depend on ongoing participation; interrupting training leads to partial loss of these adaptations, so stage-appropriate maintenance (aerobic plus RT) should be sustained long term [119, 127].

Sex-specific hormonal regulation, metabolism, and fat distribution significantly shape exercise adaptations in CKM syndrome. These variations stem from biological factors (sex chromosomes, hormonal milieu) and sociocultural influences, affecting vascular function, metabolic responses, and exercise efficacy [128].

Women generally exhibit higher rates of lipid oxidation during exercise, while men rely more on carbohydrate metabolism, a dynamic that influences exercise strategies for T2DM and MetS [99]. Estradiol provides vascular protection in premenopausal women, yet postmenopausal hormonal shifts accelerate vascular aging [59, 128, 129].

Women with T2DM experience a 3–7 times greater cardiovascular risk versus men (2–3 times), likely due to intensified systemic inflammation, poorer glycemic control, and delayed detection [130]. Traditional risks (e.g., hypertension, smoking) have a more pronounced impact on women, while female-specific conditions (polycystic ovary syndrome, adverse pregnancy outcomes, early menopause) amplify CVD susceptibility [130].

Men typically develop T2DM sooner, partly owing to elevated visceral fat accumulation and testosterone-driven metabolic profiles [106]. However, testosterone elevations in women (e.g., PCOS) increase insulin resistance, underscoring the hormonal basis of sex-specific metabolic dysfunction [106, 131].

Women often achieve stronger aerobic outcomes, such as reduced inflammation, enhanced endothelial function, and superior fat oxidation, potentially due to estrogen’s anti-inflammatory and vascular benefits [129]. Moderate-intensity continuous exercise lowers blood pressure, boosts insulin sensitivity, and enhances arterial compliance in individuals with CKD and T2DM, with women displaying higher fat oxidation and men showing greater VO₂max improvements [22, 78, 99, 110, 132, 133].

High-intensity interval training is particularly effective for men in reducing visceral fat and triglycerides, while women may favor continuous moderate-intensity training for more consistent metabolic gains [27, 96, 134, 135]. Although both sexes benefit from resistance exercise (RE) for cardiovascular and metabolic risk factors, men often exhibit more pronounced blood pressure reductions and muscle hypertrophy [36, 103, 115, 135]. In order to optimize CKM outcomes in men and women, exercise prescriptions must reflect these metabolic and hormonal distinctions. By leveraging the distinct advantages of MICE, HIIT, and RE for each sex, personalized regimens can maximize intervention efficacy.

## Stage 3: Subclinical CVD in CKM

Stage 3 CKM encompasses “Subclinical atherosclerotic CVD (ASCVD) or subclinical heart failure (HF) among individuals with excess/dysfunctional adiposity, other metabolic risk factors, or CKD.” This includes: (a) subclinical ASCVD diagnosed through coronary artery calcification or subclinical atherosclerosis by catheterization/CT angiography, (b) subclinical HF identified by elevated cardiac biomarkers (NT-proBNP ≥ 125 pg/mL, high-sensitivity troponin T ≥ 14 ng/L in women and ≥ 22 ng/L in men, high-sensitivity troponin I ≥ 10 ng/L in women and ≥ 12 ng/L in men) or echocardiographic parameters, with combined indicators suggesting highest HF risk, (c) risk equivalents of subclinical CVD such as very high-risk CKD (G4/G5 CKD or very high risk by KDIGO classification), and (d) high predicted 10-year CVD risk [1]. Section 7 provides a system-level overview of myocardial and vascular adaptations across modalities.

Exercise interventions significantly bolster cardiovascular health in individuals with subclinical ASCVD and HF by improving endothelial function, diminishing atherosclerosis biomarkers, and enhancing myocardial efficiency [3, 4, 54]. Exercise modes such as MICE, HIIE, and RE can improve FMD, stroke volume, and cardiac output, all of which promote vascular remodeling and myocardial perfusion [57, 136]. Moreover, examining the topic under a network-based analyses may demonstrate dynamics like the ones related to short sleep-restricted HIIT sessions that reorganized cardiorespiratory coupling in healthy adults, emphasizing the importance of sleep [97].

Regular aerobic exercise lowers LDL-C, curbs inflammation, and increases NO production, thereby improving vascular function and mitigating the risk of CVD progression [3, 137]. Incorporating structured exercise programs at this stage can help avert the transition to overt CVD, emphasizing a proactive stance on early cardiovascular risk management.

Epidemiological studies show an inverse association between moderate-intensity PA and coronary heart disease (CHD), with 150–300 min of MICE weekly reducing CHD risk by 14–20% [3, 4]. Even less than 150 min per week yields notable benefits [138], with MICE delivering anti-atherosclerotic, anti-ischemic, anti-arrhythmic, and antithrombotic effects, thereby significantly lowering the likelihood of cardiovascular events [3]. Executed at 50–75% HRmax (3–6 METs), MICE boosts CRF and myocardial perfusion, enhancing cardiac efficiency [139, 140].

Furthermore, MICE decreases resting heart rate, alleviates arterial stiffness, and supports vascular remodeling, mitigating age-related myocardial decline [20]. Collectively, improved blood flow, strengthened myocardial contractility, and favorable lipid profile shifts underscore MICE’s pivotal role in CHD prevention.

Vigorous-intensity physical activity (>60% functional capacity, >6 METs) effectively lowers CVD risk by boosting coronary blood flow and promoting NO-mediated vasodilation [3]. High-intensity interval training is generally more time-efficient than MICE, yielding comparable gains in VO₂max and endothelial function in less total training time [4].

High-intensity interval training can match or even surpass MICE in improving CRF, VO₂ peak, and endothelial function, particularly among post-infarction HF patients. However, HIIT’s benefits in heart failure with preserved ejection fraction (HFpEF) are inconsistent, underscoring the need for careful implementation [4, 136, 141–143].

While HIIT delivers substantial cardiovascular adaptations, safety and adherence concerns remain, especially for individuals with coronary disease, where acute event risk may be elevated compared to MICT [4, 144–146]. Accordingly, patient-specific evaluations are essential to ensure safe participation.

High-intensity interval training also promotes more pronounced cardiac morphological changes and greater peak oxygen consumption relative to MICE, sometimes leading to “Athlete’s Heart”, an advantageous adaptation typically seen in endurance athletes [20]. Research indicates that HIIT outperforms MICE in improving vascular function among those with metabolic disorders and cardiovascular disease, highlighting its efficiency as a training modality [145].

Resistance training enhances muscle strength, power, and mass while also eliciting notable cardiovascular adaptations. In contrast to aerobic exercise, RT induces left ventricular wall hypertrophy due to a higher pressure load on the heart, whereas post-exercise hypotension primarily arises from central hemodynamic shifts [20, 147]. It is well-established for improving glucose metabolism, bolstering insulin sensitivity, and reducing major CVD risk factors such as hypertension and Type 2 Diabetes. It also lowers total cholesterol and triglycerides while raising HDL-C, underscoring its importance in metabolic risk management [20, 109].

Combining aerobic and resistance training provides greater benefits than either modality alone, particularly in cardiac rehabilitation and in secondary prevention programs for individuals with established coronary artery disease [3, 4, 148]. This dual-method approach strengthens endothelial function, refines blood pressure regulation, and fosters broader metabolic adaptations. Engaging regularly in both aerobic and resistance routines reduces CVD-related mortality, advancing a holistic framework for long-term health [20].

Sex-specific physiology materially shapes training responses in subclinical CVD and warrants explicit tailoring. Influenced by hormonal levels, body composition, and metabolic profiles, these variations affect how men and women react to different training modalities [11, 149, 150]. Women with T2DM and subclinical atherosclerosis often benefit more from MICE owing to estrogen’s vascular protective effects, which enhance endothelial function, NO production, and myocardial efficiency [130, 151]. However, after menopause, accelerated vascular aging increases CVD risk despite prior estrogen-mediated protection [128]. Conversely, men typically observe larger gains in VO₂max and greater reductions in arterial stiffness with HIIE, likely due to a higher proportion of fast-twitch fibers and elevated cardiac output at maximal intensities [130, 152, 153]. Men also tend to accumulate more visceral fat, a central driver of metabolic dysfunction, whereas women store more subcutaneous fat, which may delay CVD onset [130].

During MICE, women typically experience stronger improvements in endothelial function, myocardial efficiency, and metabolic flexibility, potentially mitigating early heart failure risks [57, 58, 143, 149, 154]. By contrast, men undertaking HIIE often see greater VO₂max increases, enhanced stroke volume, and improved arterial compliance, enabling more robust cardiac function [152]. Both sexes benefit from RT, but men typically attain larger hypertrophy via testosterone-driven anabolic pathways, while women often exhibit superior muscular endurance and oxidative metabolism [11, 57, 149, 155, 156].

Women with subclinical atherosclerosis or HFpEF benefit significantly from MICE, which fosters vascular compliance and lowers inflammation [130, 157]. Men with reduced ejection fraction (HFrEF) gain more from HIIE, given its capacity to raise cardiac output and counter maladaptive left ventricular remodeling [3, 4, 142]. Resistance training remains vital for both sexes in combating sarcopenia and preserving functionality; however, women may need greater training volume to match men’s hypertrophic responses [103, 156].

Given these sex-specific differences in hormonal regulation, fat distribution, and cardiovascular function, individualized exercise prescriptions are essential. Selecting training approaches aligned with sex-based physiological adaptations can bolster cardiovascular outcomes, stall disease progression, and enhance quality of life in subclinical CVD [141, 158]. Improvements in CRF, endothelial function, and arterial compliance regress without continued training; phase-II gains should transition to a long-term maintenance program (home- or center-based) to preserve benefit [3, 4, 159].

## Stage 4: Clinical CVD in CKM

Stage 4 of CKM syndrome encompasses individuals with clinical CVD, such as coronary heart disease (CHD), heart failure (HF), stroke, peripheral artery disease (PAD), and atrial fibrillation (AF), alongside metabolic risk factors and/or CKD. This stage is subdivided into Stage 4a (no kidney failure) and Stage 4b (with kidney failure) [1]. Key system-level effects (myocardial, vascular, functional) are summarized in Sect. 7.

Supervised exercise programs are instrumental in boosting functional capacity, alleviating symptoms, and enhancing quality of life in these patients [159]. Supervised exercise therapy for PAD typically improves treadmill walking performance, with interval walking formats (e.g., 1–3 min to moderate–severe claudication / 1–2 min relief) are effective, and booster sessions help sustain gains [160]. Supervised treadmill walking to near-maximal claudication is endorsed by contemporary AHA/ACC PAD guidelines and typically elicits 6-minute walk gains of ~ 30–55 m over 3 sessions/week for 12–24 weeks [160], while structured home-based walking programs yield larger gains in six-minute walk distance over six months [161]. Practical considerations include risk stratification, progressive interval walking to moderate-to-severe claudication, and support for home-based options when access to supervised programs is limited [162–164].

Regular physical activity decreases triglycerides, blood glucose, BMI, and waist circumference while elevating HDL cholesterol. Despite these benefits, many cardiovascular patients remain inactive due to limited support or misconceptions about exercise safety [165, 166]. Even though major health organizations endorse aerobic exercise for HF patients, only about half of eligible individuals participate in cardiac rehabilitation [167].

Both HIIT and MICT improve exercise tolerance and CRF, though adherence often hinges on patient preferences and symptom profiles [25, 40, 85, 86]. Exercise augments cardiac output by initially raising heart rate and subsequently amplifying stroke volume with continued training. Endurance exercise induces concentric and eccentric cardiac remodeling, moderates sympathetic overdrive, and lowers catecholamine levels, reducing myocardial ischemia and arrhythmia risks in HF [167]. Aerobic exercise also promotes myocardial perfusion and minimizes congestion, thus improving HF outcomes and lowering hospitalization rates [168–170]. In PAD, exercise fosters collateral circulation, extending walking distances and diminishing claudication [171, 172]. In PAD, walking performance gains decline when structured programs cease; continued interval walking and periodic booster sessions help sustain distance and reduce claudication [160–163]. For stroke rehabilitation, integrating aerobic exercise enhances motor recovery, functional independence, and neural adaptation [37, 173, 174]. Structured exercise similarly reduces AF burden and heightens CRF in AF patients [37, 169, 175].

Converging population-level data support these clinical trial patterns: the Copenhagen City Heart Study indicates that higher-intensity exercise boosts longevity and mitigates chronic disease risk more effectively than exercise duration alone, although outcomes may differ across CAD and HF patients [38, 60, 140, 176]. A Cochrane Library Review further validates that both MICT and HIIT enhance CRF in CHD and HF, with HIIT conferring superior VO₂peak improvements in HF with HFrEF. By contrast, MICT and HIIT benefit HF with HFpEF comparably, without a single clearly superior method [139, 141, 142].

Moderate-intensity continuous training is widely employed in cardiac rehabilitation due to its proven effectiveness in supporting weight loss, reducing cardiovascular risk, and promoting overall heart health [166]. Although MICT enhances CRF, its effect on VO₂peak may be less pronounced over short periods than that of HIIT. Nevertheless, MICT’s favorable safety profile and broad applicability often make it the modality of choice for individuals with lower fitness or elevated cardiovascular risk [165].

Moderate-intensity continuous training can still significantly improve VO₂peak, although some research suggests that HIIT may yield superior outcomes when total energy expenditure is matched [136]. Structured MICT, lasting up to 60 min per session, three to five times weekly, has been shown to increase functional capacity, lessen dyspnea, and enhance quality of life among heart failure patients [168]. It remains fundamental in cardiac rehabilitation, with substantial evidence underscoring its contributions to symptom management and sustained cardiovascular health [167].

In stroke survivors, a six-month study comparing HIIT and MICT found both produced similar improvements in VO₂peak, physical activity levels, and mental health, reaffirming the safety and clinical value of each [152]. Within rehabilitation settings, MICT is frequently paired with resistance training to optimize patient outcomes, offering a well-rounded approach to advancing functional capacity and overall wellness [37, 177]. As a cornerstone of rehabilitation programs, MICT remains a crucial strategy for improving cardiovascular function, reducing morbidity, and enhancing quality of life. While HIIT may provide more rapid VO₂peak gains for specific populations, the recognized safety, accessibility, and durable benefits of MICT reinforce its critical role in exercise-based interventions for those with cardiovascular disease.

High-intensity interval training efficiently enhances cardiovascular fitness, metabolic health, and vascular function, particularly in individuals with CAD and HF [142, 165, 166]. Compared to MICT, HIIT delivers larger gains in VO₂peak and CRF over shorter durations, largely due to intensified oxidative metabolism [165]. HIIT also benefits lipid profiles by boosting HDL and reducing LDL, reinforcing its role in lowering cardiovascular risk [136].

In CAD patients, HIIT may surpass MICT for cardiovascular fitness, yielding greater VO₂max increases and lowering resting heart rate [178, 179]. Enhanced CRF and VO₂peak, key mortality predictors in CAD, are linked to improved vascular homeostasis, heightened NO production, and elevated endothelial shear stress [48, 147]. In acute myocardial infarction (AMI) patients with preserved systolic function, both low-volume (LV-HIIT) and high-volume (HV-HIIT) protocols improve vascular homeostasis and substantially reduce oxidized LDL (ox-LDL), underscoring HIIT’s potential for mitigating atherosclerosis [165, 180]. Nonetheless, rigorous patient evaluation and close supervision remain crucial in cardiac rehabilitation settings due to inherent cardiovascular risks [165].

Preoperative HIIT has also demonstrated advantages by improving multiple cardiorespiratory fitness metrics, including VO₂peak, six-minute walk test (6MWT) distance, and peak power output, while cutting postoperative complications by 56% [181]. However, variations in anaerobic threshold responses highlight the necessity of individualized protocols to maximize benefits. While these findings are not specific to CKM populations, they underscore HIIT’s potential for rapidly improving cardiorespiratory fitness pre-procedurally. However, translating this approach to high-risk CKM patients would necessitate careful individualized risk assessment and supervision, as high-intensity protocols may be contraindicated in certain cases. Prehabilitation within CKM Stage 4 can be targeted to upcoming cardiovascular care. In peripheral artery disease (PAD), short supervised walking programs that progress to near-maximal claudication yield clinically meaningful gains in treadmill performance and 6-minute walk distance over 12–24 weeks, providing a practical template that can be deployed before and after revascularization. Within CKM Stage 4 populations, short supervised walking programs in PAD produce 6-minute walk gains over 12–24 weeks and inform peri-procedural preparation as well as post-procedure rehabilitation [161]. In heart failure with preserved ejection fraction, supervised exercise training improves cardiorespiratory fitness and functional capacity, supporting secondary prevention pathways [159]. In chronic kidney disease, structured aerobic plus resistance training improves endothelial function and arterial stiffness surrogates, complementing standard care [119]. A pragmatic prehabilitation pattern is 3–5 days/week individualized aerobic work (including interval components) plus ~ 2 days/week resistance training; disease-specific recommendations are summarized elsewhere in Stage 4 and in Table 2 [38]. Supervised exercise likewise increases cardiorespiratory fitness in HFpEF and improves vascular indices in CKD, linking prehabilitation to disease-specific secondary prevention pathways [38, 119, 159].

A systematic review and meta-analysis of heart failure with HFpEF and reduced HFrEF reported that HIIT can outperform MICT in raising VO₂peak and left ventricular ejection fraction (LVEF). However, when contrasted with RT, no significant differences emerged, indicating the need for further investigation into optimal modalities for HFpEF [182]. Some findings suggest combining HIIT and RT may yield even greater physiological adaptations, but limited sample sizes and diverse methodologies necessitate additional research [183].

High-intensity interval training has proved safe and effective in cardiac rehabilitation for CAD, substantially increasing VO₂peak and potentially reducing premature mortality [146]. Among HF patients, it enhances peak VO₂, showing more pronounced benefits in older individuals and those with a BMI ≤ 28, although it imparts fewer gains in ventilatory efficiency (VE/VCO₂) [142]. Notably, HIIT did not outperform MICE in raising heart rate (HR) and heart rate variability (HRV) post-ST-elevation myocardial infarction (STEMI). MICE delivered superior outcomes for HR, HRV, and heart rate recovery (HRR). Even so, HIIT significantly improved exercise capacity relative to MICT and standard physical activity, displaying comparable safety [38, 184, 185]. Overall, HIIT stands as a potent, time-efficient training modality in CAD, HF, and post-MI recovery, but diligent oversight and patient-specific assessments are essential to ensure safety and sustainable adherence.

Heart failure patients often experience skeletal muscle dysfunction that lowers strength, diminishes functional capacity, and curtails exercise tolerance. Resistance exercise, particularly when combined with endurance training, has been shown to counter these limitations by boosting VO₂peak, walking capacity, and muscle strength, making it crucial in HF rehabilitation [186]. Since muscle atrophy is common in HF, RE is vital for maintaining muscle mass, enhancing strength, and improving overall exercise tolerance, making the implementation of proper biomechanics for foundational movements crucial for safety and efficacy [187]. Research has revealed marked gains in both lower- and upper-body muscle strength, along with improved cardiorespiratory fitness following structured RE [168]. Further underscoring its value, resistance training substantially elevates VO₂peak, a key predictor of long-term survival and cardiovascular health in HF [155].

Individual functional capacity, clinical status, and comorbidities must be evaluated when prescribing RE for HF patients to ensure safety and maximize efficacy [139]. Rehabilitation guidelines typically classify RT intensity as low (20–30% 1RM), moderate (40–60% 1RM), or high (80% 1RM), with 8 to 15 repetitions per set, 1 to 3 sets per session, and 2 to 3 sessions weekly, adapted to the patient’s needs [127, 168]. Resistance training significantly enhances both isokinetic and 1RM strength in HF, improving physical function and exercise capacity. Moreover, RT has been associated with superior functional outcomes, including higher VO₂peak and six-minute walk distance, reflecting greater endurance and mobility [155, 168].

Beyond building muscle strength, resistance training advances cardiorespiratory fitness and may aid in managing complex cardiovascular conditions like left ventricular assist device (LVAD) dependence and spontaneous coronary artery dissection (SCAD) [156]. Incorporating RE into a comprehensive cardiac rehabilitation plan fosters functional independence and a better quality of life for HF patients [139]. When paired with endurance training, resistance exercise remains a foundational strategy to optimize cardiovascular health and address the physical limitations tied to HF.

Sex differences in symptom presentation, disease course, and exercise adaptations in chronic cardiovascular conditions warrant individualized exercise strategies. Women with CHD often derive larger relative gains in endothelial function and aerobic capacity from MICE, likely owing to estrogen-mediated nitric oxide modulation, whereas men typically achieve more substantial increases in lean mass and strength from RE [130, 135, 155]. Adherence to physical activity guidelines remains lower among women, particularly after stroke, highlighting the need for interventions aimed at improving participation and long-term compliance [174].

In older populations with comorbidities such as obesity, diabetes, and sarcopenia, combining aerobic and resistance training yields notable improvements in physical function, balance, and cardiovascular health [188]. For those with advanced cardiovascular conditions, characterized by diminished functional capacity, compromised vascular dynamics, and increased inflammation, exercise remains essential for secondary prevention [139, 159, 189]. Moderate-intensity continuous exercise is especially effective at enhancing endothelial function, boosting left ventricular ejection fraction, and improving aerobic capacity while simultaneously reducing arterial stiffness and systemic inflammation in PAD and heart failure [37, 83, 139, 154, 159, 166, 171, 190]. High-intensity interval exercise can offer comparable or even superior VO₂peak and vascular benefits compared to MICE, with the added benefit of time efficiency. However, men often display more pronounced cardiac remodeling and left ventricular hypertrophy under high-intensity protocols, whereas women exhibit stronger endothelial adaptations [37, 83, 154, 159, 166, 171, 190, 191]. Both MICE and HIIE are safe and effective for chronic cardiovascular conditions, but sex-specific physiological differences, personal preferences, functional capacity, and comorbidities warrant tailored prescriptions [141, 178].

In addition to building muscle strength, RE promotes insulin sensitivity and functional recovery in heart failure, stroke, and atrial fibrillation [159, 168, 169, 171]. Among stroke survivors and individuals recovering from ischemic stroke or transient ischemic attack (TIA), a six-month program blending HIIT and MICT produced comparable gains in CRF, PA, and mental health, suggesting flexibility in program design [152]. Future work should further investigate sex-specific exercise prescriptions in individuals with CKM syndrome, particularly among high-risk cardiovascular populations. Leveraging digital health solutions to bolster adherence and incorporating sex-specific strategies into rehabilitation may improve patient outcomes and help mitigate inequities in cardiovascular care.

## Cross-cutting physiological systems: summary by mechanism and outcome

To reduce redundancy while preserving the stage-based structure, we synthesize here the principal exercise effects by physiological system and provide typical effect sizes and minimum effective durations While the stage-based model provides a clinical framework, the underlying physiological benefits of exercise are systemic. To complement the stage-based sections and reduce redundancy, Table 2 summarizes typical effect sizes and minimum effective durations by exercise modality.

### Carbohydrate metabolism

Combined (or concurrent) aerobic and resistance training consistently produces the most significant improvements in glycemic control, including reductions in HbA1c. The HIIT has also been shown to be a potent stimulus for improving glycemic markers and aerobic capacity in patients with type 2 diabetes [8, 24, 63, 75, 76, 89, 112, 121, 123].

### Lipid metabolism

Aerobic exercise is a cornerstone for managing dyslipidemia. Its benefits, including reductions in triglycerides and modest increases in high-density lipoprotein cholesterol, are well-documented in systematic reviews, with the magnitude of the effect often scaling with the total weekly volume and energy expenditure of exercise [16, 23].

### Vascular function

Both MICT and HIIT are effective at improving endothelial function, often measured by flow-mediated dilation. Regular aerobic exercise has been shown to improve vascular health independent of baseline cardiovascular risk [54, 57, 87, 90, 122].

### Myocardial function

In cardiac rehabilitation settings, particularly for patients with coronary artery disease or heart failure, interval training often demonstrates superior gains in peak oxygen uptake compared to volume-matched MICT [136, 142, 154, 184–190].

### Renal outcomes

Exercise is a critical, though underutilized, therapy in chronic kidney disease. Structured programs are associated with improved clinical outcomes, enhanced vascular health, and may slow disease progression through mechanisms including reduced inflammation and improved mitochondrial function [6, 35, 119].

### Body composition

Aerobic training (both MICT and HIIT) is a primary intervention for reducing visceral adipose tissue. Resistance training is essential for preserving or increasing lean muscle mass, which in turn improves basal metabolic rate and insulin sensitivity [30, 64, 79, 84, 127].

**Table 2:** Modality-specific effects—typical magnitudes and minimum effective durations

Outcome	Modality and dose	Typical magnitude and duration
VO2peak in CAD.	HIIT 3 sessions/wk, 4–12 wk.	Absolute increase ~ 1.5-3.0 mL·kg^-1·min^-1 over matched MICT; supervision recommended (70, 142, 169, 191).
Glycemic control.	Concurrent aerobic + RT (moderate-to-high intensity, ≥ 70% 1-RM for maximal HbA1c effect), 3–5 sessions/wk, 8–16 wk.	HbA1c reduction about 0.3–0.7% points; fasting glucose decreases small to moderate (97, 120).
Vascular function.	Aerobic training 3–5 sessions/wk, 8–12 wk.	FMD + 1–3% points; MICE may offer longer-lasting FMD improvements than HIIE; PWV reductions typically require > = 8–12 wk (54, 57, 58, 67, 89).
Lipids.	Aerobic training 150–300 min/wk.	Triglycerides decrease small to moderate; HDL-C increase small; dose-dependent (16)
Visceral adipose tissue.	Aerobic training of moderate to vigorous intensity, 8–16 wk.	Modest to moderate VAT reduction; dose dependent (8, 76, 127, 140).
Hepatic fat.	HIIT or MICT, 8–12 wk.	Liver fat reduction often independent of weight loss (5, 63, 64).
Albuminuria / eGFR.	Aerobic or concurrent training, 8–24 wk.	Albuminuria often decreases; structured programs may help slow CKD progression; eGFR response is variable by stage; monitor kidney function (6, 35).
6 MW distance in PAD.	Home-based walking or supervised treadmill, 12–24 wk.	+ 30 to + 55 m in 6MWD typical; supervised programs improve treadmill distance more (74).

Values represent typical ranges from randomized trials and meta-analyses; individual responses vary with baseline status, adherence, and concomitant therapies

Abbreviations: MICT, moderate-intensity continuous training; HIIT, high-intensity interval training; CR, cardiac rehabilitation; VO₂peak, peak oxygen uptake; FMD, flow-mediated dilation; HOMA-IR, homeostatic model assessment of insulin resistance; VAT, visceral adipose tissue; IHTG, intrahepatic triglyceride; eGFR, estimated glomerular filtration rate; 6MWD, 6-minute walk distance; PAD, peripheral artery disease; HDL-C, high-density lipoprotein cholesterol; PWV, pulse wave velocity; HbA1c, glycosylated hemoglobin A1c; RT, resistance training; MICE, moderate-intensity continuous exercise; CKD, chronic kidney disease

## Limitations and scope

This review follows the American Heart Association framework for CKM staging, which defines the construct and guides stage-specific interpretation of exercise effects [1, 2] and it is not free of limitations. As a narrative review, its primary scope is to provide a broad, stage-specific qualitative synthesis rather than a systematic, quantitative analysis. We integrate randomized trials and meta-analyses across varied cohorts and comparators, which introduces heterogeneity that precludes a single, definitive quantitative estimate across all endpoints. Divergent pooled results for adiposity and fitness when comparing HIIT with MICT illustrate this point [92, 93], and vascular outcomes vary by population and protocol across syntheses of endothelial function and arterial stiffness [90, 91]. We did not perform a systematic search or formal quality assessment of all included studies, which precludes definitive quantitative summaries or a formal meta-analysis. This is a particularly salient point in exercise science, where the credibility, quality, and replicability of findings, especially those related to HIIT, are topics of active academic debate [25, 85, 86, 113, 144, 183].

Furthermore, the clinical application of these findings is highly dependent on long-term patient adherence as vascular and metabolic adaptions regress with detraining, which remains a significant challenge [24, 88]. While this review outlines the efficacy of various modalities, their real-world effectiveness is often constrained by low participation and high dropout rates. This adherence challenge is a central question in the literature, particularly for high-intensity protocols, which may not be associated with higher long-term adherence despite claims to the contrary [25, 163, 164, 174]. These adherence challenges are amplified in CKM-relevant populations, such as PAD populations [163, 172], stroke survivors [174], and due to systemic issues such as limited availability, awareness, and utilization of supervised exercise therapy, that create further hurdles to implementation [164].

## Conclusion

Exercise remains a vital yet underutilized intervention in CKM syndrome management, providing stage-specific advantages that counter metabolic dysregulation, vascular dysfunction, and inflammation. This review underscores the distinct benefits of MICE/MICT, HIIE/HIIT, and RE/RT across CKM stages, reducing visceral adiposity in Stage 1, improving endothelial function and glycemic control in Stage 2, bolstering cardiovascular resilience in Stage 3, and optimizing cardiac rehabilitation outcomes in Stage 4.

Despite these documented benefits, adherence to exercise often presents a challenge, particularly among individuals with advanced CKM complications [16]. Importantly, many of these exercise-induced adaptations are transient and regress upon cessation (detraining), which makes sustained adherence a central determinant of long-term CKM outcome [88, 96, 160, 161]. Lack of structured exercise guidance, minimal patient engagement, and limited clinical integration pose substantial barriers. Overcoming these hurdles demands a multidisciplinary strategy that unites exercise specialists, physicians, and digital health technologies to create personalized, sustainable interventions [4, 5].

Additionally, sex-based differences in exercise responses call for further study, as hormone levels, fat distribution, and metabolic adaptations influence effectiveness in men and women [28, 96]. Future research should explore optimal training protocols for varied CKM populations, utilizing novel methodologies like Network Physiology to better characterize the integrated effects of stressors and exercise on cardiometabolic interactions and overall responsiveness [27, 40, 97].

Incorporating exercise into CKM clinical practice necessitates a paradigm shift, moving beyond pharmacological reliance toward comprehensive, multimodal treatments targeting CKM’s underlying causes. Digital health tools, wearable devices, and AI-based exercise monitoring can improve adherence and refine exercise prescriptions [4, 8]. By adopting a stage-specific, personalized exercise strategy, clinicians can enhance patient outcomes, reduce CKM-associated complications, and ultimately advance global cardiometabolic health.

## Data Availability

No datasets were generated or analysed during the current study.
